# Combined free flap of muscle-sparing *latissimus dorsi* and serratus anterior in the repair of child’s traumatic foot injury: a case report

**DOI:** 10.11604/pamj.2024.48.91.43952

**Published:** 2024-07-09

**Authors:** Thomas Albert, Stéphane Guero, Clément Deranque, Pascal Rousseau

**Affiliations:** 1 Institut de la Main Nantes-Atlantique, Santé Atlantique, Avenue Claude Bernard, Saint-Herblain, France,; 2 Institut de la Main, 22 rue Georges Bizet, Hôpital Necker Enfants Malades, Université Paris Descartes, 149 rue de Sevres, Paris, France,; 3 Clinique du Tertre Rouge, Pôle Santé Sud, 62 rue de Guetteloup Le Mans, Mans, France,; 4 Service de Chirurgie Plastique, Esthétique et Reconstructrice, Centre Hospitalier Universitaire d'Angers, 4 rue Larrey, Angers, France

**Keywords:** Flap, microsurgery, foot, reconstruction, case report

## Abstract

Multi-tissue injuries to the foot are common in the pediatric population. Microsurgical repairs are part of the therapeutic arsenal in pediatric reconstructive surgery. We report the case of a 4-year-old boy involved in a lawnmower accident resulting in complete amputation of the hallux, soft tissue damage, and exposure of the calcaneus and first metatarsal. A combined free flap repair using muscle-sparing latissimus dorsi and serratus anterior was performed. The patient was reviewed at 3 months and 1 year with radio-clinical and podoscopic examination. Weight bearing on the foot and on the flap was completely restored without skin fragility. Aesthetics were assessed using a numerical scale and foot function using the American Orthopaedic Foot and Ankle Society (AOFAS) and Foot and Ankle Outcome Score (FAOS) scores. The results of these scores were good, with a clear improvement at 1 year. Repair of the traumatic foot in children requires a robust surgical strategy to restore function and aesthetics to this complex organ. Our combined free flap of muscle-sparing latissimus dorsi and serratus anterioris are the only ones described in the literature. It appears to be a reliable treatment option, with no morbidity and good functional results.

## Introduction

Lawnmower accidents are frequent domestic accidents in the pediatric population [1]. They are more likely to affect young boys and are often the cause of serious multi-tissue traumas. Repair must meet a functional requirement that lasts throughout growth. It must restore function with the possibility of wearing shoes. The aesthetic aspect is also important, as there is a risk of after-effects which can be a source of mental distress, particularly in adolescence. Trauma to the foot and ankle are the most frequently encountered injuries in lawnmower accidents, after the hand. The consequences of traumatic injuries to the lower limb are all the more worrying when they occur in a young person and affect the distal region [2].

The value of microsurgery in pediatric reconstructive surgery was demonstrated later than in adults. While the first free transfers were successfully performed in adults in the 1960s, it was not until 1975 that the first pediatric free flap was performed on a 4-year-old boy [3]. With advances in microsurgery and the advent of supermicrosurgery in the 1990s, the size of vessels in children is no longer an obstacle to the creation of free flaps. Trophic sequelae of the sole of the foot, toes, and heel are frequent and difficult to resolve. There is no consensus on microsurgical repair of the traumatic foot in children. The use of a combined free flap of muscle-sparing *latissimus dorsi* (MSLD) and *Serratus anterior* (SA) could represent an interesting option in the repair of the child's foot.

## Patient and observation

**Figure 1:**
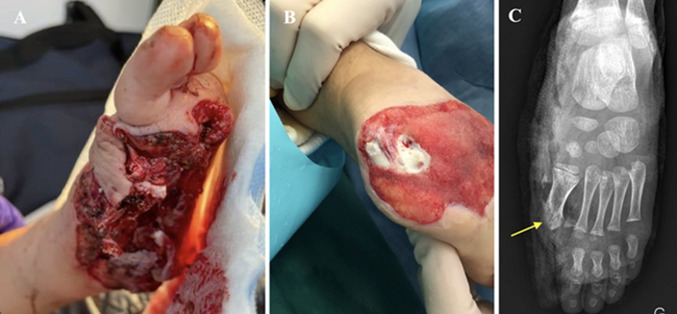
initial trauma: A) severe damage with soft tissue defect of the plantar, medial, and posterior sides of the left foot; B) bone exposure of the calcaneus after negative pressure wound therapy; C) frontal X-ray view of the foot showing trans-metatarsal amputation of the hallux with bony exposure of the head of the first metatarsal (yellow arrow)

**Patient observation, clinical findings, and diagnosis assessment:** a 4-year-old boy, with no medical history, was treated following a domestic lawnmower accident in the summer of 2022. Full trimming and negative pressure wound therapy (NPWT) were initially instituted. The size of the loss of substance was estimated at 66 cm^2^. The trauma resulted in damage to the left foot with transmetatarsal amputation of the hallux, exposing the head of the first metatarsal and the posterior and plantar surface of the calcaneus. X-rays revealed these bone lesions (Figure 1 (A, B, C)).

**Figure 2:**
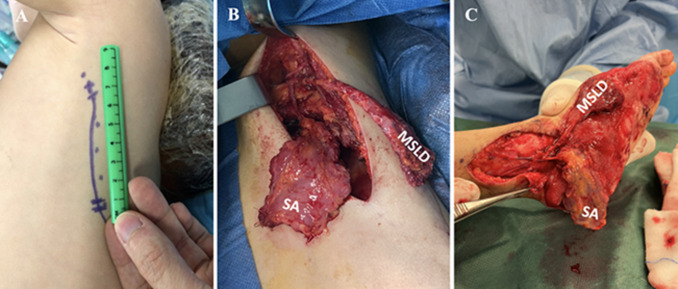
surgical procedure: A) lateral left thoracic incision (measured at 8 cm); B) harvesting of the combined free flap of muscle-sparing *latissimus dorsi* and serratus anterior; C) positioning and arteriovenous anastomoses of the flap

**Therapeutic intervention:** a combined free muscle flap of MSLD and SA was used to cover the first metatarsal and the posterior and plantar surface of the calcaneus. Preoperative computed tomography angiography (CTA) showed that all three vascular axes in the leg were patent. The patient was positioned supine with a positioning block behind the scapula to allow easy access to the harvest site. The lateral thoracic incision was measured at 8 cm (Figure 2 (A, B, C)). The operation was carried out in two teams, one team harvesting the flap and the other preparing the recipient site. The vascular pedicle contained the thoracodorsal artery and vein. They were dissected to their origin at the level of the subscapular artery. Three digitations of the SA were harvested. A portion of the LD was harvested as an anterior strip using the muscle-sparing technique from the descending branch of the thoracodorsal artery [4]. The thoracodorsal nerve was preserved in situ to avoid functional sequelae. Vascular anastomoses at the recipient site were performed with 10/0 nylon using a termino-terminal suture on the posterior tibial artery and the great saphenous vein. Xylocaine was used intraoperatively to prevent vasospasm. The quadrangular shape of the SA was used to cover the calcaneus and the MSLD was used to cover the medial aspect of the foot and the head of the first metatarsal. The cold ischemic phase time was 48 minutes and surgery time took 6 hours. The foot was immobilized postoperatively in a posterior ankle splint. No intra- or postoperative complications were reported. Intraoperative anticoagulation with heparin was used. The patient was able to go back home 10 days after the surgery.

The flap was then grafted with a thin mesh skin graft. The post-operative care was provided in a physiotherapy and rehabilitation center with orthopaedic insoles.

**Figure 3:**
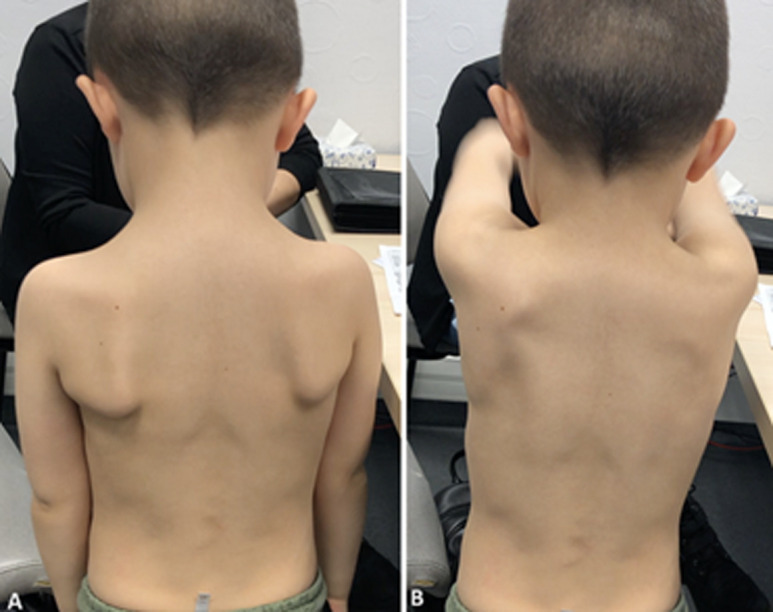
A, B) clinical exam of the shoulder at 1 month (note the absence of winging scapula)

**Figure 4:**
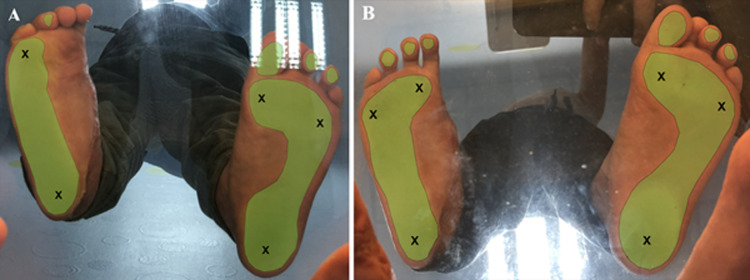
podoscopic exam, the black crosses represent the weight-bearing bony points and the green area represents the footprint: A) at 3 months; B) at 1 year (note the absence of anteromedial weight-bearing bony point at 1 month and its return at 1 year after surgery)

**Follow-up and outcomes:** at 3 months, healing was complete with a hypertrophic scar on the foot. The child and his mother assessed the aesthetic appearance of the foot as modest at 5/10. On the other hand, the lateral thoracic scar, opposite the donor site, showed no abnormality. The lateral shape of the thoracic figure was preserved. Examination of the shoulder showed full mobility and no winging scapula (Figure 3 (A, B)). Barefoot walking revealed a limp with hyper-weight-bearing on the lateral edge of the foot, an absence of anteromedial weight-bearing, and a pathological supinated position (Figure 4 A). Running and sporting activities were not possible. The AOFAS and FAOS scores were 63% and 78% respectively.

**Figure 5:**
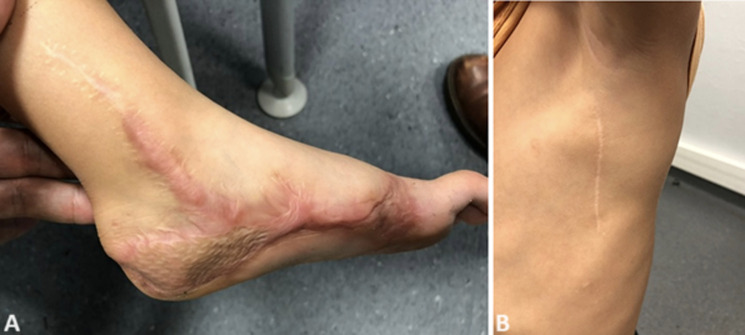
A, B) appearance of the foot and donor site at 1 year

**Figure 6:**
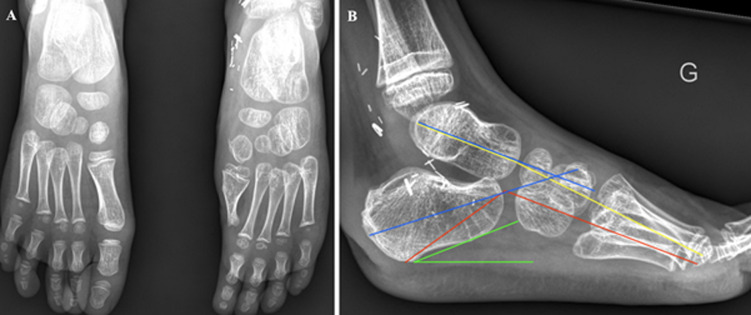
X-ray at 1 year: A) frontal view of both feet; B) weight-bearing lateral view of the left foot with measurement of foot statics parameters (Djian-Annonier angle in red, Méary-Toméno line in yellow, calcaneal slope in green, talocalcaneal divergence angle in blue; all measurements are normal)

At 1 year, the anteromedial weight-bearing foot was returned and the supinated position was corrected (Figure 4B). The hypertrophic scar showed signs of regression (Figure 5 (A, B)). The aesthetic appearance of the foot was judged to be very satisfactory (8/10). The lateral thoracic scar still showed no abnormality. Unipedal stance was possible, as were toes and heels walking. Walking and running were possible without assistance or a limp, and the patient was able to get back to his sporting activities (skiing, swimming, and football). AOFAS and FAOS scores increased to 92% and 98% respectively. X-rays showed harmonious growth of the first metatarsal, and angle measurements, performed on profile view X-rays, did not reveal any impairment of foot full weight-bearing (Figure 6 (A, B)).

**Patient perspective:** the patient considers himself healed and parents consider their child healed. They are satisfied with the complete medical and surgical management.

**Informed consent:** the patient and her parents have been informed of the publication of this case, why the case was special, and the authors' intention to publish it.

**Patient consent:** the patient and her parents have given their consent for their images and other clinical information to be published in the journal.

## Discussion

Despite the advent of fascio-cutaneous perforator flaps in the late 2000s, we still believe that the use of muscle flaps in foot repair surgery remains appropriate. Firstly, muscle flaps have a potential role in limiting infection [5]. They are reliable flaps, with constant vascular pedicles and good diameters. Therefore, they are of particular interest in the pediatric population, where the small size of the vessels can make microsurgical procedures difficult. The latissimus dorsi (LD) flap and the anterolateral thigh flap (ALT) have similar coverage capacities in terms of area and could be in competition in their use. However, it is interesting to note that the diameter of the thoracodorsal artery is twice that of the perforating arteries that vascularize the anterolateral thigh skin.

Free flap surgery in children appears to be superior to locoregional flaps in terms of aesthetic and functional results [6]. In addition, locoregional vascularization of the distal third of the lower limb is more insecure, which increases the interest in using free flaps. The advantage of microsurgery in children is the usual absence of cardiovascular risk factors. Free flaps in children have fewer complications and enable them to resume their activities more quickly. The use of LD for muscle sparing, described by Tobin *et al*. in 1980, reduces donor site morbidity by preserving muscle function, reduces seroma formation, and appears to be suitable for the pediatric population [4]. The SA has a microsurgical success rate comparable to that of LD and is better in children than in adults [7]. This thin muscle is well adapted to the heel and arch of the foot. This is why Duteille *et al*. have made it a reference flap in foot repair surgery [8]. Muscle flaps have a good ability to adhere to the recipient site, unlike fascio-cutaneous flaps which can induce a soaping effect [9]. As the muscle atrophies, it moulds itself to the deep contours of the bone tissue. The muscle would provide superior support to that obtained with fascio-cutaneous flaps in the weight-bearing areas of the foot and would retain its ability to be sensitive to pressure [10]. The use of muscle flaps in weight-bearing anatomical areas subject to mechanical friction, such as the foot, seems to us to be an interesting solution. On the other hand, the absence of superficial skin sensation may be a factor in the skin ulceration frequently observed, even if the sensation of deep pressure is present. Children are certainly less aware of the risks of walking on insensitive ground and their cooperation, even with the help of parents, may not be as strong as in adults. Thus, it is possible that support on the reconstructed foot in children is prolonged compared with adults. In our study, podoscopy showed hyper weight bearing on the traumatized foot. The capacity of the muscles to resist pressure is interesting but remains limited.

The use of NPWT as a waiting solution before flap coverage seems to be an interesting solution. But its use alone does not seem to be a good solution because of the poor quality of healing.

## Conclusion

Our combined free flap of MSLD and SA used for microsurgical repair of the traumatic foot in children is the only one described in the literature. No study has evaluated the function and aesthetics of a reconstructed traumatic foot with objective scores in the paediatric population. With excellent results on function and aesthetics, this combined free flap seems to have a place in the reconstruction of the traumatic foot in children.
